# ZNF545 suppresses human hepatocellular carcinoma growth by inhibiting NF-kB signaling

**DOI:** 10.18632/genesandcancer.137

**Published:** 2017-03

**Authors:** Weili Yang, Shuai Yang, Meiying Zhang, Dan Gao, Tao He, Mingzhou Guo

**Affiliations:** ^1^ Department of Gastroenterology and Hepatology, Chinese PLA General Hospital, Beijing, China; ^2^ Medical College of NanKai University, Tianjin, China

**Keywords:** ZNF545, hepatocellular carcinoma, NF-kB signaling, DNA methylation, epigenetics

## Abstract

Hepatocellular carcinoma (HCC) is one of the most common cancers and the second leading cause of cancer related death worldwide. ZNF545 is located in the chromosome 19q13.13, which is frequent loss of heterozygosity in human astrocytoma. Methylation of ZNF545 was found frequently in a few kinds of cancers. While the function of ZNF545 in human HCC remains unclear. The purpose of this study is to explore the function and mechanism of ZNF545 in human HCC. Restoration of ZNF545 expression suppressed cell proliferation, migration and invasion, induced G1/S arrest and apoptosis in SNU449 and Huh7 cells. Further study suggested that ZNF545 suppressed HCC cell growth by inhibiting NF-kB signaling. These results were further validated by siRNA knocking down technique in ZNF545 highly expressed HXBF344 cells. In vivo, ZNF545 suppressed tumor growth in SNU449 cell xenograft mice. In conclusion, ZNF545 suppresses human HCC growth by inhibiting NF-kB signaling.

## INTRODUCTION

Hepatocellular carcinoma (HCC) is the fifth most common cancer worldwide and the second cause of cancer-associated death [1, 2]. Hepatitis B virus (HBV) and Hepatitis C virus (HCV) infection constitutes the most important cause of HCC. Alcohol abuse and dietary exposure to aflatoxin B contamination are also involved in HCC carcinogenesis[3]. Until recently, only four genes (TP53, CTNNB1, ARID1A and AXIN1) were found mutated more than 10% in HCC [3, 4]. While, aberrant epigenetic changes were found frequently in human HCC. DNA methylation was found involved in various cancer-related signaling pathways, including Wnt, p53, and TGF-β signaling [5-9].

As the critical functions in signaling cascades, the zinc finger protein family is recognized as one of the most important transcriptional factors. Zinc fingers perform crucial roles in maintaining physiological processes from prokaryotes to eukaryotes [10]. Zinc-finger protein 545 (ZNF545) is a transcription factor, which was reported to be involved in different cancers [11-13]. The function of ZNF545 in human HCC remains obscure. This study is mainly to investigate the function of ZNF545 in human HCC.

## RESULTS

### ZNF545 suppresses cell proliferation, induces G1/S arrest and apoptosis in human HCC cells

**Figure 1:**
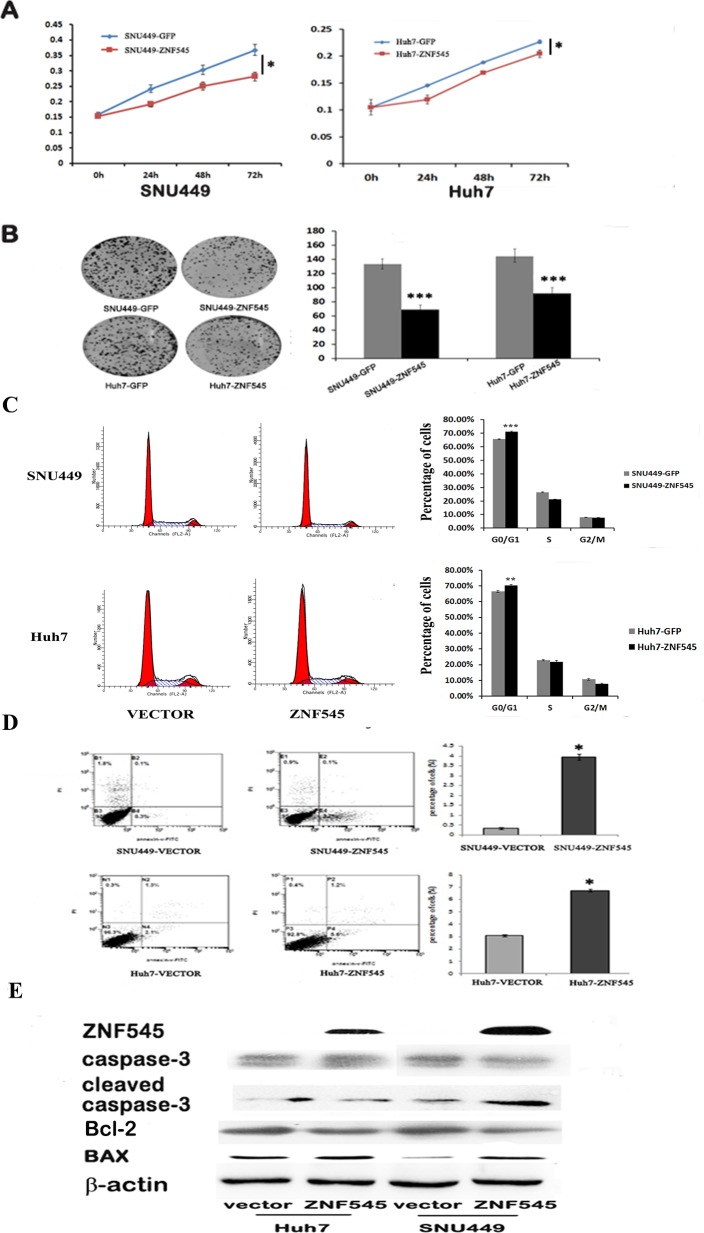
ZNF545 suppresses cell proliferation, induces G1/S arrest and induces cell apoptosis in human HCC cells (A) Growth curves represent the effects of unexpressed and re-expressed ZNF545 in SNU449 and Huh7 cells analyzed by the MTT assay. (B) Colony formation results show that the colony number was reduced by re-expression of ZNF545 in SNU449 and Huh7 cells. The average number of tumor clones is represented by the bar diagram. (C) Cell phase distribution in ZNF545 unexpressed and re-expressed SNU449 and Huh7 cells. The ratios are presented by bar diagram. (D) Flow cytometry results show the role of ZNF545 in apoptosis in SNU449 and Huh7 cells. Each experiment was repeated in triplicate. *P<0.05. **P<0.01. ***P<0.001. (E)Western blots show the effects of ZNF545 on caspase-3, cleaved caspase-3, Bcl2 and BAX in SNU449 and Huh7 cells. β-actin: internal control.

In previous study, we found ZNF545 was frequently methylated in human HCC and the expression of ZNF545 was regulated by promoter region methylation [14]. While the function of ZNF545 in human HCC remains unclear. To explore the effect of ZNF545 on HCC cell viability, MTT assay was employed. As shown in Figure1A, the OD value was 0.3033 ± 0.006 *vs.* 0.2506 ± 0.0005 in SNU449 cells and 0.2267 ± 0.003 *vs.* 0.2043 ± 0.007 in Huh7 cells before and after restoration of ZNF545 expression. The OD value was significantly different (all *P*<0.05). The cell viability was suppressed by ZNF545 in HCC cells. Colony formation assay was used to evaluate the role of ZNF545 in cell proliferation. The colony numbers were 133.25 ± 7.27 *vs.* 68.75 ± 10.53 in SNU449 cells and 144 ± 11.11 *vs.* 91.25 ± 8.26 in Huh7 cells before and after re-expression of ZNF545. The colony number was significantly reduced after re-expression of ZNF545 in HCC cells (all *P*<0.001, Figure 1B). These results demonstrate that ZNF545 suppresses the proliferation of HCC cells.

The role of ZNF545 on cell cycle was analyzed by flow cytometry. As shown in Figure 1C, the distribution of cell phases were 66.47 ± 0.66% *vs.* 70.40 ± 0.45% in G0/G1 phase, 22.82 ± 0.33% *vs.* 21.74 ± 0.95% in S phase and 10.71 ± 0.58% *vs.* 7.86 ± 0.47% in G2/M phase in ZNF545 unexpressed and re-expressed Huh7 cells. The G0/G1 phase was increased significantly (*P*<0.01). In SNU449 cells, the cell phases distribution were 65.47 ± 0.32% *vs.* 71.26 ± 0.29% in G0/G1 phase, 26.49 ± 0.34% *vs.* 21.10 ± 0.32% in S phase and 7.87 ± 0.074% *vs.* 7.64 ± 0.046% in G2/M phase before and after restoration of ZNF545 expression. The G0/G1 phase was significantly increased (*P*<0.001).

The effect of ZNF545 on cell apoptosis was analyzed by flow cytometry. The percentage of apoptotic cells was 0.33 ± 0.06% *vs.* 3.93 ± 0.15% in SNU449 cells and 3.1 ± 0.1% *vs.* 6.73 ± 0.12% in Huh7 cells before and after re-expression of ZNF545. Cell apoptosis was increased significantly by ZNF545 in SNU449 and Huh7cells (both *P*< 0.05, Figure 1D). The role of ZNF545 in cell apoptosis was further validated by western blotting. The levels of caspase-3 and Bcl2 were reduced and the levels of cleaved caspase-3 and BAX were increased after re-expression of ZNF545 in SNU449 and Huh7 cells (Figure 1E).

### ZNF545 inhibits cell migration and invasion in HCC cells

**Figure 2:**
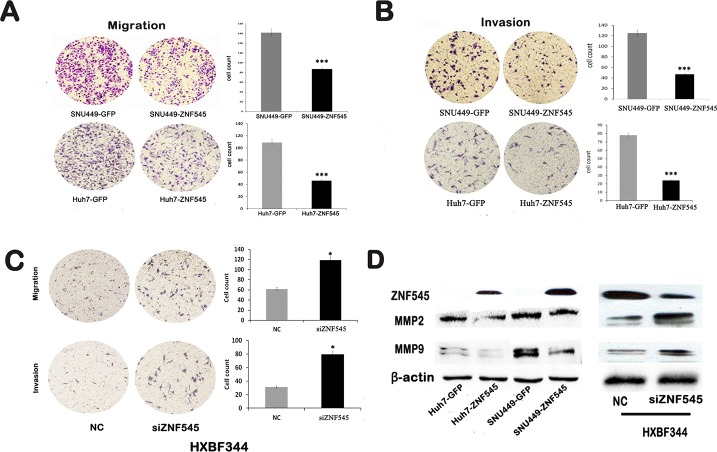
ZNF545 inhibits cell migration and invasion in HCC cells (A) Cell migration experiment in SNU449 and Huh7 cells before and after re-expression of ZNF545. The average number of migration cells is presented by a bar diagram. (B) Cell invasion experiment in SNU449 and Huh7 cells before and after re-expression of ZNF545. The average number of invasion cells is presented by a bar diagram. (C) Cell migration and invasion experiments with and without knockdown of ZNF545 in HXBF344 cells. The average number of migration and invasion cells is presented by a bar diagram. Each experiment was repeated in triplicate. *P<0.05. **P<0.01. ***P<0.001. (D) The expression of MMP2 and MMP9 was detected by Western blot. β-actin: internal control. NC: normal control.

To evaluate the effect of ZNF545 on HCC cell migration and invasion, the transwell assay was performed. As shown in Figure 2A, the migration cell numbers were 161.75 ± 18.84 *vs.* 87.25 ± 6.08 in SNU449 cells and 109 ± 8.98 *vs.* 46.25 ± 5.32 in Huh7 cells before and after re-expression of ZNF545. The migration cell number was significantly reduced after re-expression of ZNF545 (all *P*<0.001). The invasion numbers were 121 ± 5.91 *vs.* 47 ± 6 in SNU449 cells and 78 ± 9.52 *vs.* 24 ± 2.45 in Huh7 cell before and after re-expression of ZNF545 (Figure 2B). The number of invasion cells was significantly reduced (all *P*<0.001) after re-expression of ZNF545. These results suggest that ZNF545 inhibits HCC cell migration and invasion. The roles of ZNF545 in cell migration and invasion were further validated by analyzing the expression levels of MMP2 and MMP9 with western blotting. As shown in Figure 2D, the expression levels of MMP2 and MMP9 were reduced after re-expression of ZNF545 in SNU449 and Huh7 cells.

The effects of ZNF545 on migration and invasion were further validated by siRNA technique in ZNF545 highly expressed HBX344 cells. The migration cell numbers were 61.8 ± 15.08 *vs.* 118.9 ± 18.64 and the invasion cell numbers were 31.2 ± 3.3 *vs.* 79.73 ± 12.95 in HXBF344 cells before and after the knockdown of ZNF545. The invasion and migration cells were increased significantly after knockdown of ZNF545 in HXBF344 cells (all *P*<0.05, Figure 2C). As shown in Figure 2D, the expression levels of MMP2 and MMP9 were increased after knockdown of ZNF545 in HXBF344 cells. Above results suggest that ZNF545 suppresses HCC cell migration and invasion in HCC.

### ZNF545 suppresses HCC cell growth by inhibiting NF-kB signaling

**Figure 3:**
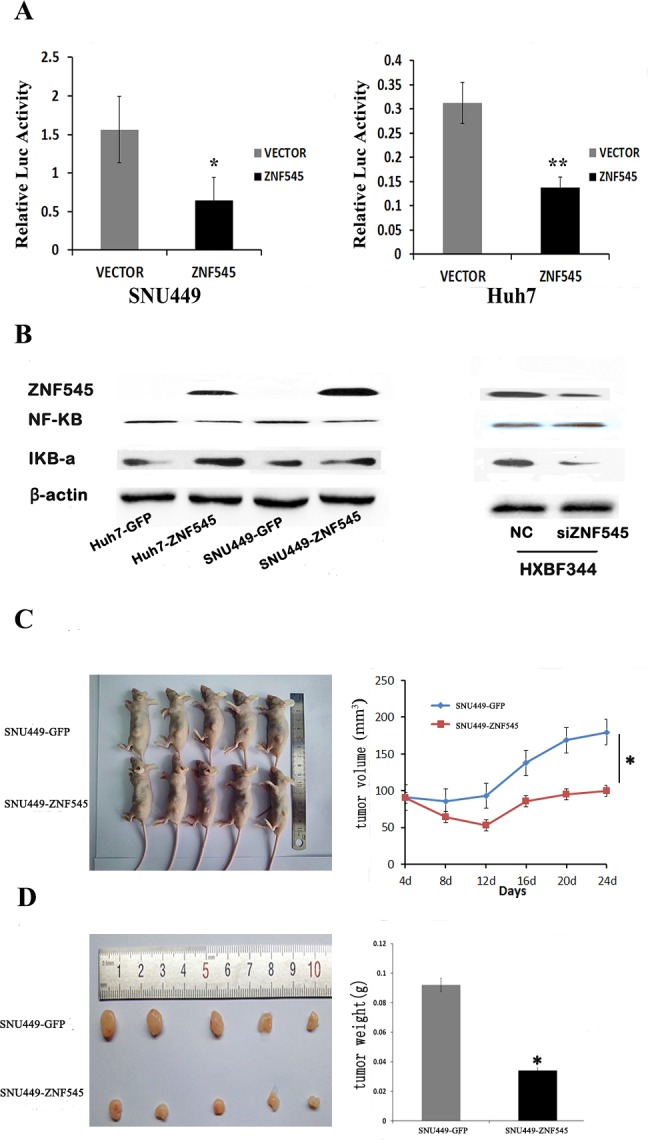
ZNF545 suppresses HCC cell growth by inhibiting NF-kB signaling and ZNF545 suppresses human HCC cell tumor growth in xenograft mice (A) SNU449 and Huh7 cells were co-transfected with 3×kB-luc, pcDNA3.1 (+)-ZNF545 and vectors. Forty-eight hours later, luciferase assays were performed. Each experiment was repeated in triplicate. *P<0.05. **P<0.01. ***P<0.001. (B) The levels of NF-kB (p65) and Ikβ-α were detected by Western blot in SNU449 and Huh7 cells; the levels of NF-kB (p65) and Ikβ-α were detected by Western blot after knockdown of ZNF545 in HXBF344 cells. β-actin: internal control. NC: normal control. (C) Subcutaneous tumor growth curves for xenograft mice burdened with SNU449 cells in which ZNF545 is unexpressed and re-expressed at different times. (D) Tumor weight in nude mice at the 24th day after inoculation with SNU449 cells in which ZNF545 is unexpressed and re-expressed. Bars indicate mean of five mice. *P<0.05.

ZNF545 was found to suppress gastric cancer cell growth by inhibiting NF-kB signaling [10]. To determine whether the NF-kB signaling pathway is regulated by ZNF545 in human hepatocellular carcinoma, a dual-luciferase reporter assay was employed. The relative luciferase activity was 1.56 ± 0.43 *vs.* 0.64 ± 0.30 in SNU449 cells and 0.31 ± 0.043 *vs.* 0.14 ± 0.022 in Huh7 cells (Figure 3A). The activity of NF-kB was significantly inhibited by ZNF545 in SNU449 and Huh7 cells (both *P* <0.05). To further verify the mechanism of ZNF545 in HCC, the effect of ZNF545 on NF-kB signaling was analyzed in HCC cells by Western Blot. The expression levels of Ikβ-α were increased, and the expression levels of NF-kB were reduced after re-expression of ZNF545 in SNU449 and Huh7 cells (Figure 3B). These results indicate that NF-kB signaling is inhibited by ZNF545 in HCC. To further validate the effect of ZNF545 on NF-kB signaling, siRNA knockdown technique was employed. As shown in Figure 3B, the levels of Ikβ-α were reduced, and the levels of NF-kB were increased after knockdown of ZNF545 in ZNF545 highly expressed HXBF344 cells. The results further suggest that ZNF545 suppresses HCC cell growth by inhibiting NF-kB signaling.

### ZNF545 suppresses human HCC cell tumor growth in xenograft mice

To further investigate the roles of ZNF545 in human HCC, ZNF545 unexpressed and re-expressed SNU449 cell xenograft mouse models were employed. The tumor volumes were 179.67 ± 71.54 mm^3^ *vs.* 95.5 ± 57.68 mm^3^ in ZNF545 unexpressed and re-expressed SNU449 cell xenograft mice (Figure 3C). The tumor weights were 0.092 ± 0.054g *vs.* 0.034 ± 0.009g in ZNF545 unexpressed and re-expressed SNU449 cell xenograft mice (Figure 3D). The tumor volumes and weights were reduced after re-expression of ZNF545 (all *P* < 0.05). These results demonstrate that ZNF545 suppresses human HCC *in vivo*.

## DISCUSSION

Transcription factors play a central role in regulating gene expression, and therefore coordinate a plethora of biological processes, including differentiation, development, metabolism, apoptosis and autophagy [15-19]. Based on different DNA binding motifs, transcription factors can be mainly categorized into classical zinc fingers, homeodomains, and basic helix-loop-helix [20-22]. Classical zinc finger containing proteins (ZNFs) form the largest family of sequence-specific DNA binding protein, which are encoded by 2% of human genes[23, 24]. More and more evidences have proved that aberrant expression of ZNF proteins contribute to tumorigenesis in various cancers. Different types of zinc finger motifs show great diversity of biological functions. About a third of ZFPs contain the kruppel-associated box (KRAB) motif, and more than half of KRAB-ZFPs are clustered in the region 19q13[25]. KRAB-ZFPs play an important role in regulating cell proliferation, apoptosis, differentiation, and tumorigenesis[26]. ZNF545 is also a member of KRAB-ZFPs and located in the chromosome 19q13.13, which is frequent loss of heterozygosity in human astrocytoma [27, 28]. ZNF545 was found frequently methylated in different cancers [11-14]. ZNF545 inhibits multiple myeloma tumor growth by activating p53 pathway[11]. While the function and the mechanism of ZNF545 in HCC remains unclear. In this study we found that ZNF545 inhibits cell proliferation, induces G1/S arrest and apoptosis in human HCC. In addition, ZNF545 suppresses human HCC cell migration and invasion. In vivo, ZNF545 suppresses HCC cell tumor growth in xenograft mice. These results suggest that ZNF545 is involved in HCC development and metastasis. As reported in other cancers, ZNF545 suppresses HCC growth by inhibiting NF-kB signaling. ZNF545 is a tumor suppressor in human HCC. In conclusion, ZNF545 suppresses HCC growth by inhibiting NF-kB signaling.

## MATERIAL AND METHODS

### Expressional vector construction and transfection

The CDS region of ZNF545 was amplified by RT-PCR and cloned into the pcDNA3.1 (+) expression vector. Primer sequences are as follows: 5′-CGCGGATCCGCCACCATGGCCCTTCGAT CAGT-3′ (F) and 5′-CCGCTCGAGTTAGATTTTTACATTAT-3′(R). ZNF545 cDNA was then subcloned into the pLenti6-GFP lentivirus expression vector. ZNF545 expressing lentiviral or pLenti6-GFP were packaged using the ViraPower^TM^ lentiviral expression system (Invitrogen, San Diego, CA, USA). Lentivirus was added to the supernatant of SNU449 and Huh7 cell culture medium, and ZNF545 stably expressed cells were selected by blasticidin (2μg/ml, Invitrogen, USA).

### Cell viability assay

Cells were seeded into 96-well plates at 2000 cells/well, and the cell viability was measured by the MTT assay at 0, 24, 48 and 72h (KeyGEN Biotech, Nanjing, China). Absorbance was measured on microplate reader (Thermo Multiskan MK3, MA, USA) at a wavelength of 490nm.

### Colony formation assay

Cells were seeded into 6-well culture plates at a density of 200 cells/well in triplicate and cultured for 2 weeks. Cells were then fixed with 75% ethanol for 30 minutes, stained with 0.2% crystal violet (Beyotime, Nanjing, China) for 20 minutes and counted.

### Flow cytometry analysis

For cell cycle analysis, SNU449 and Huh7 in which ZNF545 was unexpressed and re-expressed were fixed with 70% ethanol and stained with 50 mg/ml propidium iodide (KeyGEN Biotech, Jiangsu, China). The cells were then sorted by a FACS Caliber (BD Biosciences, San Jose, CA) and analyzed by the Modifit software (Verity Software House, ME, USA).

For apoptosis analysis, the Annexin V-FITC/PI Apoptosis Detection Kit (KeyGen Biotech, Nanjing, China) was used according to manufacturer's instructions. Each sample was analyzed by flow cytometry with a FACScan Flow Cytometer (Becton-Dickinson Biosciences, Mansfield, MA).

### Transwell assay

SNU449 and Huh7 in which ZNF545 was unexpressed and re-expressed were suspended in serum-free medium. Cells (2×10^5^) were placed into the upper chamber of an 8-um pore size transwell apparatus (Corning, NY, USA) and incubated for 30 hours. Cells that migrated to lower surface of the membrane were stained with crystal violet and counted in three independent high-power fields (×200). For invasion analysis, SNU449 and Huh7 in which ZNF545 was unexpressed and re-expressed (2×10^5^) were seeded into the upper chamber of a transwell apparatus coated with extracellular matrix (ECM) gel (BD Biosciences, San Jose, CA) and incubated for 48 hours. Cells that invaded the lower membrane surface were stained with crystal violet and counted in three independent high-power fields (×200). The migration and invasion assay of HXBF344 cells (1×10^5^) were performed as mentioned above, and the incubated time were 10 hours and 48 hours respectively.

### Luciferase reporter assays

Dual-luciferase assays were carried out according to manufacturer's protocol (Promega, WI, USA) as described previously[29, 30]. Cells were seeded in 12-well culture plates and were co-transfected with 0.75ug of the 3×kB-luc reporter construct and the pcDNA3.1 (+)-ZNF545 plasmid or the pcDNA3.1 (+) plasmid. pRL-SV40 (1ng) was used as an internal control. The total amount of nucleotide was kept constant by supplementing with pcDNA3.1. Cells were harvested forty-eight hours after transfection, Luciferase activity was analyzed with the Dual-Luciferase Reporter Assay System ( Promega, WI, USA ).

### Western blot

Western blot was performed as described previously [5]. Antibodies were diluted according to manufacturer's instructions. The primary antibodies used were as follows: ZNF545(Santa Cruz Biotechnology, USA), MMP2(Protein Tech Group, Chicago, IL, USA), MMP9(Protein Tech Group, Chicago, IL, USA), caspase-3(Protein Tech Group, Chicago, IL, USA), cleaved caspase-3(Protein Tech Group, Chicago, IL, USA), BAX(Protein Tech Group, Chicago, IL, USA), Bcl-2(Protein Tech Group, Chicago, IL, USA), Ikβ-α (Protein Tech Group, Chicago, IL, USA), NF-kB (Protein Tech Group, Chicago, IL, USA) and β-actin (Bioworld Tech, MN, USA).

### SiRNA knockdown assay

SiRNA knockdown assay was performed according to the manufacturer's instructions. The sequences of siRNA targeting ZNF545 and RNAi Negative Control Duplex are as follows: siRNA duplex (sense: 5′-CUGGGAUGCUUCAUUUUATT-3′; antisense: 5′-UAGAAAUGAAGCAUCCCAGTT-3′); RNAi negative control duplex (sense:5′-UCCUCCGAACGUGUCACGUTT-3′; antisense:5′-CGUGACACGUUCGGAGAATT-3′)(Gene Pharma Co, Shanghai, China).

### ZNF545 unexpressed and re-expressed SNU449 cell xenograft mouse model

Animal experiments were reviewed and approved by the Chinese PLA General Hospital Experimental Animal Committee. ZNF545 unexpressed and ZNF545 stably expressed SNU449 cells were injected subcutaneously into the dorsal left side of four–week-old female Balb/c nude mice (n = 10) weighing 12 to 18 g. Tumor volumes were measured every 4 days for 20 days starting 4 days after implantation. Tumor volumes were calculated according to the formula: V = L × W^2^/2, where V represents volume (mm^3^), L represents largest diameter (mm), and W represents smallest diameter (mm). Mice were sacrificed after 20 days.

### Statistical analysis

SPSS 17.0 software was used for data analysis. All data are presented as means ± standard deviation (SD) of at least three independent experiments and analyzed using the student's t test. *P*<0.05 is regarded as a significant difference.
